# Treatment of Diaphyseal Fractures of the Femur in Paediatric Age Group: A Comparative Study of Locking Compression Plate Versus Titanium Elastic Nailing System (TENS)

**DOI:** 10.7759/cureus.28924

**Published:** 2022-09-08

**Authors:** Sagar Venkataraman, Prabhu Ethiraj, Arun H Shanthappa, Kishore Vellingiri

**Affiliations:** 1 Department of Orthopaedics, Sri Devaraj Urs Medical College, Sri Devaraj Urs Academy of Higher Education and Research, Kolar, IND

**Keywords:** comparative study, paediatric age, locking compression plate, tens, diaphyseal fractures

## Abstract

Background: Diaphyseal femur fractures are commonly seen in the paediatric age group as there is an increase in the incidence of road traffic accidents. Titanium elastic nailing system (TENS) and plating are the common methods used for paediatric long bone fracture fixation. The purpose of our study was to evaluate and compare functional and radiological outcomes of paediatric femur diaphyseal fractures treated with locking compression plates and with TENS.

Methods: Our study included 59 patients diagnosed with femur shaft fracture. Twenty-eight patients included in group one underwent open reduction and internal fixation with locking compression plates and 31 patients in group two underwent open reduction/closed reduction with intramedullary TENS. All post-operation patients were evaluated at four, eight, 10, 12, 16, 20, 24, and 36 weeks. The functional outcome was assessed based on the Flynn scoring system and radiological union based on fracture union on X-ray.

Results: We analyzed our data using the Flynn scoring system. In group one, out of 28 cases treated with locking compression plates, 25 (89%) were excellent, two (7.5%) were satisfactory, and one (3.5%) was poor. In group two, out of 31 cases treated with intramedullary TENS, 26 (83.8%) were excellent and five (16.2%) were satisfactory. In our study, the average union time in group one was 11.4 weeks and in group two was 14.41 weeks. Fracture union was 100% in both groups.

Conclusion: In our study, we noted that the union of the femur shaft was early with the use of locking compression plates. In TENS, there was less intraoperative blood loss, very minimal postoperative scar, and less soft tissue damage. Also, implant removal was easier compared to locking compression plates.

## Introduction

Diaphyseal femur fractures are commonly seen in the paediatric age group as there is an increase in incidence due to road traffic accidents [1]. It is more commonly seen in males [2]. Children of less than five years have good remodelling potential. So fractures in this age group can be managed conservatively with traction and hip spica application [3]. Children of more than five years with displaced femur shaft fracture require operative management to prevent complications like limb length discrepancy, non-union, malalignment, and growth disturbances [4,5]. Operative management is also preferred for early ambulation and shorter hospital stay to prevent psychological and social effects which are often associated with prolonged non-operative treatment methods [5]. Various operative treatment and fracture fixation methods are available based on patient age group, fracture pattern, associated injuries, and socio-economic factors [5].

Operative treatment and fracture fixation methods include open reduction plate fixation with locking compression plate, dynamic compression plates and bridge plating, closed reduction/open reduction with intramedullary titanium elastic nailing system (TENS), stainless steel nailing, and locked intramedullary nailing; external fixators for open fractures are used to manage femur shaft fractures in children [3,5]. TENS and plating are the common methods used for paediatric long bone fracture fixation. TENS is recommended in the paediatric age group between five to 11 years [4,5]. TENS has advantages over plating as it reduces intraoperative blood loss, has shorter operative time, is comparatively less painful, and needs shorter hospital stay [4]. TENS in selected paediatric femur diaphyseal fractures is reasonably effective [6]. TENS is suitable for middle one-third femur shaft fracture and simple fractures.

In severely comminuted and long oblique fractures, other methods of fracture fixation should be considered for better outcomes [7]. Complications like varus and valgus malalignment, recurvatum or ante-recurvatum, limb length discrepancy, pain at nail entry site have been noted with TENS [8]. Paediatric femur shaft fractures treated with open reduction and plating method offered faster union rates and early weight bearing with minimal complication rates compared to intramedullary TENS [9]. In comminuted fracture, unstable fracture, and diaphyseal femur fracture, submuscular plating has given good results compared to elastic intramedullary nailing [10]. The plating method offered good rotation stability of fracture fragments. Malalignment and limb length discrepancy were minimal in the plating method compared to intramedullary TENS [11]. In the literature, various methods are used to treat paediatric diaphyseal femur fracture. The purpose of our study was to evaluate the functional and radiological outcomes of paediatric femur diaphyseal fracture treated with locking compression plate and with TENS.

## Materials and methods

The study was conducted at R.L. Jalappa Hospital & Research Centre, Kolar, Karnataka, India, after obtaining ethical clearance from institution. This was a prospective study that included cases between January 2018 to December 2020. A total of 59 patients who met the inclusion criteria were included in our study and divided into two groups. In group one, patients treated by open reduction and internal fixation with locking compression plates were included. In group two, patients treated with open reduction/closed reduction with intramedullary TENS were included. Among 59 patients, 28 patients were in group one and 31 patients were in group two. All the cases were operated on by trained orthopaedic surgeons in their speciality. We analyzed data by collecting essential/required details like preliminary data, history, clinical examination findings, operative notes, preoperative and postoperative radiographs, and follow-up notes from patient records. Postoperatively, the patient was followed up at four, eight, 10, 12, 16, 20, 24 and 36 weeks. 

The functional outcome was assessed based on the Flynn scoring system [12]. The radiological union was assessed based on fracture union on radiographs. Patients in the age group five to 14 years who sustained closed displaced fracture shaft of femur and open fractures of type I, type II, and type IIIA based on Gustilo-Anderson classification were included in this study. Patients in the age group five to 14 years who had pathological femur factures, non-union at fracture site, femur fracture with associated neurovascular injury, and crush injury of leg were excluded. Data was entered in Microsoft Excel (Microsoft Corporation, Redmond, Washington, United States) and analyzed using Stata Statistical Software, Version 12 (Released 2011; StataCorp., College Station, Texas, United States). Categorical variables such as gender, affected side, mode of injury, type of fracture, complications, and functional outcomes were summarized as proportions (percentages). Age was summarized as mean and standard deviation. Fracture union time in weeks was summarized using mean and standard deviation as well as median (interquartile range). These proportions were compared between the locking compression plate group and the intramedullary TENS group using the Chi-square test. Continuous variables such as the age of the patient and fracture union time in weeks were compared using unpaired t-test and Mann-Whitney U test. A p-value less than 0.05 was considered statistically significant.

## Results

Out of 59 patients, 28 patients were operated on with open reduction and internal fixation with locking compression plates (group one) and the other 31 patients with open/closed reduction with intramedullary TENS (group 2). Among the study group, 39 were males, and 20 were females. There was a male preponderance. The youngest age among patients was six years old and the oldest age was 14 years old. The average age was 9.81 years. Right side femur shaft fractures 31 (53%) were more compared to left side femur shaft fractures 28 (47%), as shown in Table 1.

**Table 1:** Demographic data TENS = titanium elastic nailing system; SD = standard deviation

	Locking Compression Plate Group (N=28)	Intramedullary TENS Group (N=31)	Total (N=59)	P-value
Mean (SD) age in years	10.42 (2.04)	9.25 (2.48)	9.81 (2.34)	0.054
Sex				
Male	18 (64.3%)	21 (67.7%)	39 (66.1%)	0.779
Female	10 (35.7%)	10 (32.3%)	20 (33.9%)	
Leg Affected				
Right	15 (53.6%)	16 (51.6%)	31 (52.5%)	0.880
Left	13 (46.4%)	15 (48.4%)	28 (47.5%)	

Considering the mode of injury, road traffic accident accounted for 58%, other injuries like fall during playing sports were seen in 29%, fall from height accounted for 10%, and assault accounted for three percent. For types of fractures, 22 (37.3%) fractures were transverse, 13 (22%) fractures were comminuted, 18 (31%) fractures were oblique, and six (10%) fractures were spiral, as shown in Table 2.

**Table 2:** Comparison of types and modes of injury between two study groups TENS = titanium elastic nailing system; RTA = road traffic accident

	Locking Compression Plate Group (N=28)	Intramedullary TENS (N=31)	Total (N=59)	P-value
Type of fracture				
Comminuted	7 (25.0%)	6 (19.4%)	13 (22.0%)	0.855
Oblique	9 (32.1%)	9 (29.0%)	18 (30.5%)	
Spiral	2 (7.1%)	4 (12.9%)	6 (10.2%)	
Transverse	10 (35.7%)	12 (38.7%)	22 (37.3%)	
Mode of Injury				
RTA	17 (60.7%)	17 (54.8%)	34 (57.6%)	0.621
Self-fall	5 (17.9%)	4 (12.9%)	9 (15.2%)	
Fall from height	2 (7.1%)	4 (12.9%)	6 (10.2%)	
Sports injury	4 (14.3%)	4 (12.9%)	8 (13.6%)	
Assault	0	2 (6.4%)	2 (3.4%)	

Table 3 shows comparisons of fracture union in weeks and complications between the two study groups. In our study, the average union time in group one was 11.4 weeks and in group two was 14.41 weeks. Early complications in the form of superficial infection in group one were four and in group two were two. Late complications in the form of thigh pain in group one were five and in group two were four. Cases of knee stiffness in group one were two and in group two were zero, and cases of delayed union in group one were one and in group two were four.

**Table 3:** Comparisons of fracture union and complications between the two study groups SD = standard deviation; IQR = interquartile range; TENS = titanium elastic nailing system

	Locking Compression Plate Group (N=28)	Intramedullary TENS (N=31)	Total (N=59)	P-value
Fracture union in weeks				
Mean (SD)	11.42 (2.33)	14.41 (2.04)	13.0 (2.63)	<0.001
Median (IQR)	11.5 (9.5 – 13.5)	14 (12 – 16)	13 (12 – 14)	<0.001
Less than 12 weeks	20 (71.4%)	9 (29.0%)	29 (49.1%)	0.005
12 - 17 weeks	7 (25.0%)	18 (58.1%)	25 (42.4%)	
More than 18 weeks	1 (3.6%)	4 (12.9%)	5 (8.5%)	
Complications				
No complications	18 (64.3%)	22 (71.0%)	40 (67.8%)	0.296
Thigh pain	5 (17.9%)	4 (12.9%)	9 (15.3%)	
Superficial Infection	4 (14.3%)	2 (6.5%)	6 (10.2%)	
Delayed union	1 (3.6%)	4 (12.9%)	5 (8.5%)	
Knee stiffness	2 (7.1%)	0	2 (3.4%)	

**Figure 1:**
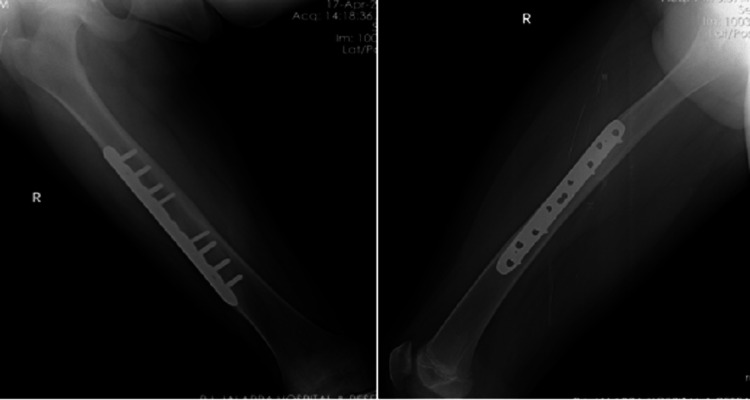
Femur shaft fracture operated with locking compression plating

**Figure 2:**
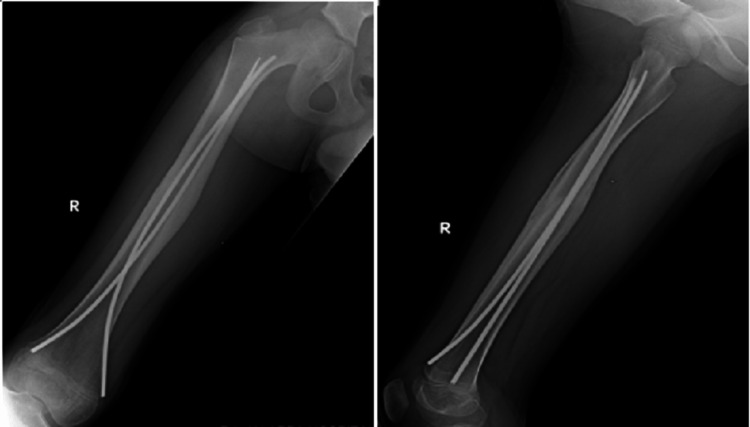
Femur shaft fracture operated with titanium elastic nailing system

The functional outcomes were evaluated based on the Flynn scoring system. In group one, out of 28 cases treated with locking compression plates, 25 (89.3%) were excellent, 2 (7.1%) were satisfactory, and 1 (3.6%) were poor. In group two, out of 31 cases treated with intramedullary TENS, 26 (83.9%) were excellent, and five (16.1%) were satisfactory, as shown in Table 4. Radiograph of plating is shown in Figure 1 and of nailing in Figure 2.

**Table 4:** Functional outcomes based on the Flynn scoring system [12] TENS = titanium elastic nailing system

Functional Outcomes	Locking Compression Plate Group (N=28)	Intramedullary TENS (N=31)	Total (N=59)
Excellent	25 (89.3%)	26 (83.9%)	51 (86.4%)
Satisfactory	2 (7.1%)	5 (16.1%)	7 (16.1%)
Poor	1 (3.6%)	0	1 (1.7%)
Total	28 (100%)	31 (100%)	59 (100%)

## Discussion

It is important to restore normal anatomy of femur following fracture with minimal soft tissue damage and retaining vascularity and periosteal blood supply. While achieving fracture reduction, it is important to achieve compression at fracture site, maintain rotational stability, and prevent malalignment and limb length discrepancy. Any abnormality in femur will alter hip and knee range of movements. There are different modalities of treatment for treating paediatric femur shaft fracture. Individual treatment depends on patient age factor, fracture pattern, and socioeconomic status of the patient. We evaluated two different methods: group 1 was open reduction and internal fixation by plate fixation methods and group 2 was closed reduction/open reduction using intramedullary TENS. We compared functional and radiological outcomes of both the methods in paediatric diaphyseal femur fracture. In our study, functional outcome was assessed based on the Flynn scoring system. In group one, excellent results were seen in 89%, satisfactory results in 7.5%, and poor results in 3.5%. In group two, excellent results were seen in 83.8% and satisfactory results seen in 16.2%. Excellent results were seen in 67.8% and satisfactory results seen in 32.2% in plating group and in TENS group, excellent results were seen in 62% and satisfactory results in 38% [13]. Excellent results were seen in 75.5%, satisfactory outcome in 17%, and poor outcome in 7.5% of cases treated with TENS [14].

In our study, radiological union was seen in all the cases. We evaluated union rates statistically using Chi-square test. Average time for union in group one (open reduction and internal fixation by plate fixation) was 11.40 weeks, whereas, in the Abdelgawad et al. [15] study, it was 13.3 weeks. In group two (closed reduction/open reduction using titanium elastic intramedullary nailing), average time for union was 14.41 weeks, whereas in the Pogorelić et al [16] study, it was 8.5 weeks. We had five cases of delayed union out of 59 cases, which took 18-24 weeks to unite. Out of five patients, one patient was from group one and four patients were from group two. In group one, it was severe comminuted fracture which took longer time to unite. In group two patients, there was loss of fracture reduction on subsequent follow-up radiographs in three of our patients due to early weight bearing. In spite of strict instruction not to weight bear early, children started weight bearing early. And in group two, one of the patients had a comminuted fracture. All these factors lead to delayed union in group two.

In our study, fracture union in group one was early and malalignment in the forms of varus-valgus, procurvatum and recurvatum was seen in two out of 28 patients, which too was in acceptable range (less than 10 degrees). In one patient in group one, due to early weight bearing, there was an increase in malalignment, limb length discrepancy, and persistence of pain and deformity, which resulted in poor outcome. Whereas in group two patients, five patients out of 31 patients had rotational malalignment (less than ten degrees) which was seen on subsequent follow-up radiograph and four patients had limb length discrepancy of 1-2cms at the end of one-year follow-up. We noted that in our study, minimal intraoperative blood loss, minimal postoperative scar, less soft tissue damage, and easier implant removal happened in group two compared to group one but blood loss was not quantified intraoperative. The average hospital duration stay postoperatively was four to seven days in group two compared to group one where it was 10-14 days.

Limitations of the study

The current study had a number of drawbacks. This study used information from many surgeons and was conducted at a single tertiary care facility. In the preoperative and postoperative phases, the statistics were subject to fluctuation based on surgeon preference. A prospective multicenter study with a bigger sample size would do away with these limitations.

## Conclusions

In our study, we noted that radiological union of femur shaft fracture in paediatric age group was early when treated with locking compression plates compared with intramedullary nail (TENS). All fractures were united in our study group. In paediatric femur fractures treated with intramedullary TENS, there was minimal intraoperative blood loss, very minimal postoperative scar, less soft tissue damage, and easier implant removal compared to locking compression plate procedure, where blood loss was more and one more major operative procedure was required for implant removal.
